# The economic impact of COVID-19 on the creative industries: a sub-regional input–output approach

**DOI:** 10.1007/s12076-023-00329-9

**Published:** 2023-02-24

**Authors:** Matthew S. Lyons

**Affiliations:** University of Birmingham (City-REDI), Birmingham, UK

**Keywords:** Input–output, COVID-19, Creative industries, R15, C67, L82

## Abstract

The creative industries are a strategically important sector for the Cardiff Capital Region (CCR) which houses large public sector broadcasters and an ecosystem of IT and software businesses. The CCR is an administrative boundary in Wales which captures just under half of the Welsh population but over half of the Welsh economy. The pandemic and resultant lockdown restrictions have had profound impacts on the creative industries, a sector which depends heavily on in-person interaction. The creative industries are not one homogenous sector, but a collection of different activities some of which faced different supply and demand conditions due to the COVID-19 shock. To understand the impact of the shock in fine inter-industry detail and at a sub-regional scale an input–output table for the Cardiff Capital Region (the CCRC-IO) is utilized. The CCRC-IO estimates that the direct, indirect, and induced impacts of the shock see output fall by £457 m (0.53% of CCR output), GVA by £147 m (0.58% of CCR GVA) and FTE employment by 2416 (0.58% of CCR FTE). The paper finds that the economic impact of the COVID-19 shock varies considerably by both geography and sub-sector.

## Introduction

The creative industries have been seen increasingly as a priority in economic development plans at the national, regional, and sub-regional level in the UK, in view of their export potential and capacity to catalyse economic growth (HM Government 2018). At the same time the spatial focus of economic development has been shifting towards sub-regions a trend that may be set to continue as devolution is placed centrally in the UK Government’s levelling up agenda (HM Government 2022).

The Cardiff Capital Region (CCR) like other UK regions, has placed significant emphasis on the creative industries as part of its economic development strategy. The CCR is an interesting example city-region in that it houses a capital city, Cardiff and has a history of devolution in broadcasting and culture.

The COVID-19 pandemic has had significant sustained impacts on both the supply and demand sides of the global economy (Baldwin and Tomiura 2020). The creative industries have been the worst impacted sector outside of tourism and hospitality (Khlystova et al. 2022). The impact of the shock has varied across the creative industries with the film and television sub-sector particularly badly affected. The CCR has a greater presence of film and TV production than other UK city-regions (Lyons and Davies 2022) and therefore is a particularly interesting case study for evaluating the impact of the shock.

To date the evaluation of economic shocks in an IO framework in Wales has been limited to the national level with insufficient disaggregation to reveal the creative industries. This analysis seeks to understand the impact the COVID-19 pandemic has had on the CCR creative industries using a novel sub-regional IO table extended to reveal the creative sectors (Lyons 2022). The contributions of this paper are twofold. Firstly, this paper considers the impact of COVID-19 using IO at a fine geography relevant to the current policy discourse, the city-region. Secondly, the analysis focuses in on a narrow selection of industrial sectors, sub-sectors of the creative industries.

## Literature summary

Ramos et al. (2020) view COVID-19 much like a natural disaster which largely effects the demand for ‘non-essential’ goods and services. While there isn’t a settled definition of non-essential goods with some including only food, defence, and medical goods Court et al. (2021) consider the sectors that have been exposed or not exposed to lockdown restrictions. In the UK, a list of critical workers provides a guide to those sectors considered essential HM Government (2020). The creative industries were not on the critical workers list and with many activities requiring large in-person gatherings significant activity in the sector was halted during the strictest lockdown measures.

IO can be a helpful tool for evaluating demand shocks within an economy revealing inter-industry impacts throughout the local economy. There are numerous examples of the utilization of IO to model COVID-19 already in the literature (Court et al. 2021; Pichler et al. 2021; Rose et al. 2021; Guan et al. 2020). However, much of the analysis of the economic impacts of COVID-19 to date has focused on the impacts at the national level and the implications for trade and supply chains.

There has been a great deal of attention paid to the impact of COVID-19 on the creative industries as the worst impacted sector outside of tourism and hospitality, a detailed literature review of which can be found in (Khlystova et al. 2022). In the UK, the COVID-19 shock in 2020 has been most significant in creative sub-sectors dependent on large indoor gatherings: performing arts, theatre, and live music which due to lockdown restrictions and cautious consumers have seen significant falls in activity. In 2020, the film and TV sector was particularly badly affected with productions which can require many people on set shutdown (Siepel et al. 2021).

Despite this interest, there has been little analysis on the impact of COVID-19 on the creative industries using an IO framework which reflects the relatively limited application of IO in the creative industries. Part of the reason for this is that the creative industries are not typically disaggregated within national or regional tables (Throsby 2008). There has been considerable work from national governments and international organisations in recent years to improve coverage in this area (UNESCO 2013). However, in the UK IO and IO tables for Wales, the creative industries remain insufficiently disaggregated for effective analysis.

Clusters of creative industries activity are spread across the UK and are heterogeneous in their sub-sectoral composition. With the knowledge that creative industries present differently across regions and that COVID-19 affected sectors to varying degrees it is valuable to be able to see the impact of the shock at the regional level.

The use of IO at the sub-regional scale is relatively novel in the literature although growing in relevance in the UK in response to the changing scale of policy focus towards city-regions (O’Brien and Pike 2019). Despite the relative novelty there are examples of sub-regional IO in the UK. Carasscal-Incerra et al., (2021) compile a multi-region input–output table at the NUTS-2 geography for the UK. Hermannsson (2016) compiles a three-region IO table to evaluate the expenditure patterns of HEIs in the Glasgow city-region. Wingham and Hope (2019) detail the construction of an IO table for London.

## Data

### Compiling the cardiff capital region creative input–output table (CCRC-IO)

The CCRC-IO provides a detailed picture of the CCR economy with fine detail on the creative industries. The CCRC-IO model allows for the evaluation of direct, indirect, and induced impacts due to changes in the CCR economy such as the demand shocks resulting from COVID-19.

The CCRC-IO was compiled through the regionalisation and disaggregation of an unpublished input–output table for Wales base year 2018 which is detailed in full in Lyons (2022). Figure shows an overview of the process of regionalisation for the CCRC-IO.

The accuracy of the various techniques used to regionalise IO tables are an area of active debate (Lahr et al. 2020 and Flegg et al. 2021). The IO table for Wales is regionalised to the CCR by using a FLQs following the methodology from (Hermannsson 2016)^1^:

The industry demand in the Wales IO table for 2018 consists of 88 sectors which were reconciled with the 99 sectors within the UK SUT tables (ONS 2020). The CCRC-IO table seeks to provide accurate information on the creative industries with less focus on the non-creative industries sectors. Therefore, the CCRC-IO is aggregated to 20 sectors. The 20 sectors that remain are public administration, education, eight ‘parent’ sectors of the creative industries the nine sub-sectors of the creative industries (Appendix 1) and the remainder ‘all other sectors’.

Defining and measuring creative employment is a known challenge in the literature (Cruz and Teixeira 2012; Lyons 2022). For this paper the creative industries follow the definitions given by the DCMS (2016) which separate nine sub-sectors defined by four-digit SIC 2007 codes (see Table 1). The UK supply and use tables (ONS 2020) were used to construct creative industries sub-sector vectors for inter-industry spending, imports, compensation of employees and taxes (full detail in Lyons 2022).

**Table 1:** Distribution of GVA (£m) in the creative industries in the CCR 2018.* Source*: (Lyons 2022)

	GVA £ millions	GVA % of total
Craft	2.7	0.4%
Publishing	24.2	3.3%
Film, TV, video, radio and photography	119.2	16.2%
IT, software and computer services	314.4	42.7%
Architecture	46.7	6.3%
Advertising and marketing	24.5	3.3%
Design: product, graphic and fashion design	99.4	13.5%
Music, performing and visual arts	90.5	12.3%
Museums, galleries and libraries	15.4	2.1%
Total	737.1	100%

To estimate the size of the creative industries in the CCR, published GVA figures for Wales by creative industry sub-sector were used and scaled to the CCR level. DCMS (2020) estimate total GVA of all creative industries in Wales for 2018 at £1.02bn. To apportion the creative industries total across sub-sectors ONS (2017) figures were used which provide a breakdown of creative industries GVA for Wales in 2015. The two datasets provide an estimate of GVA by creative sub-sector in Wales for 2018. To apportion the £1.02bn Wales creative GVA to the CCR, ONS employment data (ONS 2021) were used to estimate the CCR share of GVA.

Table 1 shows the GVA (£ millions) by creative industries sub-sectors calculated from the detailed datasets. These figures show the two most significant sub-sectors to be IT, software and computer services and Film, TV, video, radio and photography together representing 58% of creative industries GVA in the CCR.

In the CCRC-IO table, final demand consists of consumer demand, capital expenditure, demand for government products and exports separated into three destinations—Rest of Wales, Rest of UK, and Rest of World. Final demand for non-creative sectors is calculated through the regionalisation of the Wales IO table using the FLQ approach and other secondary sources on trade data (Lyons 2022).

For the creative sectors, components of final demand are tackled separately. For example, government funding for the creative industries in the CCR is identified through financial statements and government releases. The impact of government funding is of particular relevance for non-market sectors such as Museums, Galleries and Libraries which receive a high proportion of their income from government grants and a relatively low share of income from market activity such as admission ticket sales. For commercially focused creative industries sectors such as Architecture government funding makes up a much smaller share of the sector income.

## Results

### The cardiff capital region creative economy

**Fig. 1:**
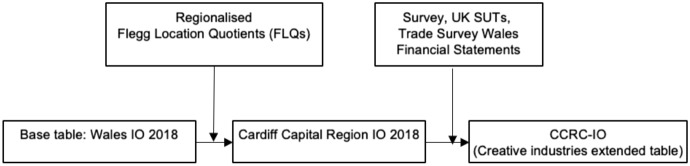
Cardiff capital region extended input–output table, regionalisation approach.* Source* (Lyons 2022)

Table 2 compares the headline figures for the economy of Wales to the economy of the Cardiff Capital Region. The table shows that the CCR contributes £88bn in output, £25bn in GVA and 419,000 FTE employment representing 51%, 39% and 51% respectively to the economy of Wales (Fig. 1).

**Table 2:** Comparing Wales to the Cardiff Capital Region: Headline figures and the creative industries breakdown. *Source* adapted from (Lyons 2022)

	Wales	CCR
Output (£ billions) GVA (£ billions) Employment (FTE)	166 64.7 816,000	86 25.2 419,000
CCR creative industries breakdown	Non-creative Industries	Creative Industries
Output (£ billions) GVA (£ billions) Employment (FTE)	86.07 24.51 408,095	2.9 0.74 11,095

Table 2 also shows the breakdown of economic activity within the CCR that is linked to the creative industries. The table shows the creative industries represent a relatively small portion of the CCR economy supporting £2.9bn in output (3.5% of CCR total) £737 million in GVA (2.9%) and sustain 11,095 FTE jobs (2.6% of all FTE employment). The CCR is hosts a significant amount of creative employment with 11,095 FTE employees representing 63% of all creative FTE in Wales (17,510 FTE). It should be noted, the labour structure of the creative industries is different to traditional economic sectors with a much higher proportion of self-employed freelance workers (Easton and Cauldwell-French 2017). As such, particularly for some sub-sectors the employment data are likely to underestimate the total number of workers.

### The COVID-19 shock in the CCR creative industries

The impact of COVID-19 has been significant and heterogeneously spread across sub-sectors of the creative industries. Depending on the stage of the pandemic and the government restrictions in place some creative businesses were unable to open or only permitted to open at restricted capacity. Some creative industries sub-sectors such as IT, software services were able to adapt more easily to home working and even saw an increase in demand for digital services (Khlystova et al. 2022). Demand side issues related to changes in consumption patterns in response to restrictions, health and economic concerns also surfaced. We follow the conclusions of Court et al. (2021) that the COVID-19 shock can be treated as a shock to the demand for non-essential goods and services.

For this paper we treat the change between 2019 and 2020 as the impact of COVID-19. However, it is important to note that in the UK 2020 is the year the country formally exited the EU and the impacts of which will have happened concurrently with COVID-19. The impacts of Brexit within the creative industries are particularly related to the labour supply (Montalto et al. 2021) effectively disaggregating the impact of Brexit is beyond the scope of this paper.

**Fig. 2:**
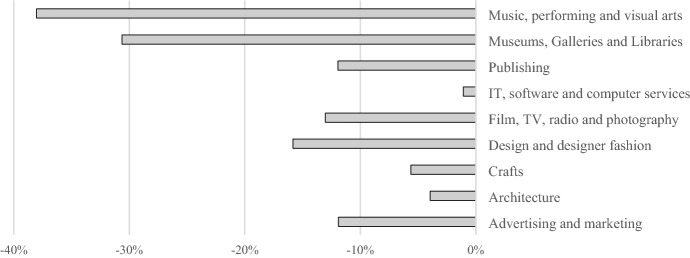
Percentage change in GVA between 2020 and 2019 for creative sub-sectors in the UK.* Source* (ONS)

To estimate the impact COVID-19 has had on the CCR creative sub-sectors final demand is changed for each sub-sector until the change in GVA matches the reported change in GVA of the nine sub-sectors between 2019 and 2020 at the UK level. UK level data is used rather than at the CCR geography as it is disaggregated beyond the two-digit SIC level to the four-digit SIC level necessary to capture creative industries sub-sectors. Figure 2 shows the change for each sub-sector. Sub-sectors that depend on in-person activity tend to have fared poorly notably, Music, performing and visual arts and Museums, Galleries and Libraries. These two sub-sectors are most closely linked to the ‘culture’ sector. Closely behind we find Design and designer fashion, an industry more dependent on goods than services and Film, TV, radio and photography a sector which depends on large crews meeting in person. There is then another group of activities which has seen a much smaller negative impact: Crafts, Architecture and IT, software and computer services. These are activities which adjust more easily to reduced in-person contact.

Table 3 shows the scenario produced for the CCRC-IO based on the GVA figures given in Fig. 2. The figures show the largest falls in final demand in percentage terms in Music, performing and visual arts and Museums, galleries and libraries. In monetary terms the falls are largest in Music, performing and visual arts and Design: product and graphic design.

**Table 3:** GVA change estimated scenarios for the COVID-19 shock in the CCR creative industries in 2020

	Final demand (£ millions)	Change in final demand (£ million)	% Change in final demand
Publishing	218	− 24.0	− 11%
Film, TV, video, radio and photography	277	− 29.2	− 11%
IT, software and computer services	940	− 7.7	− 1%
Architecture	391	− 11.1	− 3%
Advertising and marketing	163	− 13.8	− 8%
Design: product, graphic and fashion design	279	− 31.0	− 11%
Music, performing and visual arts	174	− 58.0	− 33%
Museums, galleries and libraries	67	− 19.5	− 29%

*Craft sub-sector is excluded due to low employment figures*

Table 4 shows the output from the CCRC-IO simulation which highlights the direct, indirect, and induced impact of the revenue drops estimated in the creative industries during 2020. The table shows a direct effect of the initial change in demand showing a decrease of £194 m in output, £67 m in GVA and 986 FTE jobs. The indirect effect reports to change in inter-industry activity in response to the initial shock. Induced effects are the result of the change in consumption related to the direct and indirect effects. When the total impact is considered, output drops by £457 m, GVA by £147 m and FTE employment by 2416. The impact of the drop in output in the creative industries, therefore, will not only reduce employment, output and GVA in the creative industries but, also have knock-on impacts throughout the CCR economy.

**Table 4:** CCRC-IO simulation of COVID-19 impact on the creative industries in the CCR economy in 2020

	Direct	Indirect	Induced	Total
Output £ millions	− 194.28	− 99.94	− 162.43	− 456.67
GVA £ millions	− 66.90	− 31.92	− 48.52	− 147.35
Employment (FTE)	− 986	− 642	− 788	− 2416

	Direct & indirect	Induced	Total
FTE change non-creative	− 452	− 753	− 1205
FTE change creative industries	− 1177	− 35	− 1212

The simulation indicates that the direct and indirect impacts of the shock saw 1177 FTEs lost across the creative industries, representing 72% of all lost FTE. This reflects the relatively high degree of inter-industry purchasing within the creative industries. When the induced impacts are considered, 753 FTEs (95%) were lost outside the creative industries. This is to be expected as most household consumption is spent on essential goods and services with a relatively small share of incomes spent on local creative industries outputs. When the total impact on employment is considered of the 2416 FTE lost 50.1% is found across the creative industries. The creative sub-sectors that are most significantly impacted were Film, TV, video radio and photography representing 13.6% of all FTE lost and Music, performing and visual arts representing 14.3%. This mirrors findings for other UK regions (Siepel et al. 2021).

**Fig. 3:**
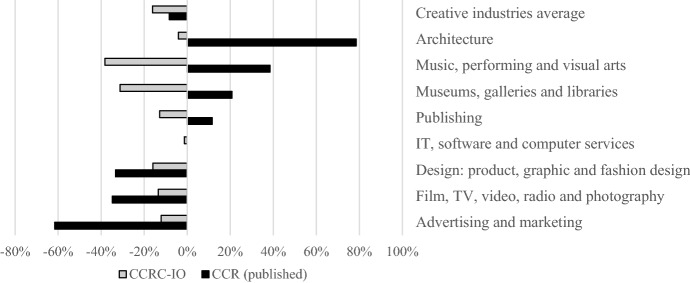
Change in FTE creative sub-sectors 2019 vs 2020 CCR published, and CCRC-IO simulated.* Source* (ONS 2021)

Figure 3 compares the change in sub-sector employment between 2019 and 2020 as published by the ONS for the CCR with the outcome of the CCRC-IO model simulation in Table 4.

The CCRC-IO figures show employment has fallen in every sub-sector reflecting the changes in GVA between 2019 and 2020. This is to be expected as the CCRC-IO is responding to the negative demand change shock. In the published employment figures the picture is more mixed with some sectors seeing a significant positive change in employment, most surprisingly in in-person focused sub-sector Music, performing, and visual arts. Architecture represents the most extreme example suggesting a near 80% increase in employment. The swing appears to be due to an outlier in the published figures for 2019 in which Architecture employment is significantly undercounted with the correction in 2020 appearing as a significant increase. The variation shows that employment figures at fine geographies and sub-sectoral level are subject to considerable noise.

There are various explanations as to why the published figures differ from the modelled estimates. The CCRC-IO assumes a fixed relationship between GVA and employment. A modelled fall in GVA leads to an expected fall in employment. However, in response to COVID-19 government interventions such as the furlough scheme disrupted the relationship between GVA and employment. As such while GVA fell, employment did not and instead a drop in GVA per employee was observed.

## Concluding remarks

The increasing importance of the creative industries for economic development in sub-regional governance has increased demand for detailed economic intelligence on the local creative economy. This paper has provided an evaluation of the economic impact of COVID-19 on creative industries sub-sectors in the CCR. The paper finds that the economic impact varies considerably by both geography and sub-sector. The CCR specialism in film & TV puts the region at greater risk than other UK regions due to the exposure the sub-sector has had to the crisis.

A further compounding factor is the reliance of the film & TV sub-sector on freelancers and casual employees. Freelancers and self-employed workers are typically employed on temporary contracts putting workers in a precarious position in a period of economic instability. A survey by Creative Cardiff (2020) into the impact of COVID-19 self-employment income support found that 60% of freelancers work had “dried up completely” and 35% reported eligibility issues for income support due to their status as recent freelancers. These impacts may have driven creative workers out of the creative labour market in the long-term presenting a risk to the sustainability and resilience of the sector already short of appropriately skilled workers (Lyons 2022).

The COVID-19 shock will affect city-regions across the UK differently depending on the character of their creative industries cluster. City-regions with a greater share of IT, software and computer services, for example, will have been less badly affected by the initial shock and will likely remain more resilient over the coming years.

Significant challenges remain for modelling impacts at the sub-regional scale most notably the difficulty in gathering timely data on sub-sectors at sub-national geographies. This analysis applies national data on the of the impact on COVID-19 based on the change in GVA between 2019 and 2020 and finds some divergence between the modelled outcomes and the outcomes found in the CCR FTE employment figures.

## Appendix

See Table

**Table 5:** Creative industries sector disaggregation in the CCRC-IO

Non-creative sectors	All other sectors Public admin Education
‘Parent’ sectors of creative industries*	Other manufacturing Publishing (remainder) Computer and related activities Other professional services Market research, advertising Other recreation, media & film
Creative industries sub-sectors**	Craft Publishing Film, TV, video, radio, and photography IT, software, and computer services Architecture Advertising and marketing Design: product, graphic and fashion design Music, performing and visual arts Museums, libraries, and galleries

^*^Parent sectors are the remainder of sectors in the 2018 Wales IO table from which the creative industries sectors were disaggregated. **As defined in DCMS (2016)

5.

## Footnotes

1 A key consideration for the FLQ approach is assigning an appropriate value for the δ parameter. Changing the δ parameter adjusts the allowance for inter-regional imports with a higher δ assuming greater imports. For this study we use a value of 0.3 (Lyons 2022).
